# Effects of matcha tea extract on cell viability and estrogen receptor-β expression on MCF-7 breast cancer cells

**DOI:** 10.1007/s00404-023-07209-z

**Published:** 2023-09-22

**Authors:** Simon Keckstein, Constantin Tilgener, Udo Jeschke, Simone Hofmann, Theresa Vilsmaier, Lucia Keilmann, Helene Heidegger, Till Kaltofen, Falk Batz, Sven Mahner, Lennard Schröder

**Affiliations:** 1 Department of Obstetrics and Gynecology, University Hospital, LMU Munich, Marchioninistr. 15, 81377 Munich, Germany; 2 Department of Urology and Transplant Surgery, Klinikum Stuttgart, Stuttgart, Germany; 3 Department of Obstetrics and Gynecology, University Hospital Augsburg, Augsburg, Germany; 4 Department of Surgery, University Hospital Regensburg, Regensburg, Germany

**Keywords:** Matcha tea extract, MTE, MCF-7, ERβ, WST-1, PCR, Western blot

## Abstract

**Purpose:**

In the following work, we investigated the effect of matcha green tea extract (MTE) on MCF-7 breast cancer cell viability and estrogen receptor-beta expression (ERβ).

**Methods:**

MCF-7 cells were stimulated with MTE at concentrations of 5 and 10 µg/ml. Cell viability was assessed using a water-soluble tetrazolium assay (WST-1 assay) after an incubation time of 72 h. ERβ was quantified at gene level by real-time polymerase chain reaction (PCR). A western blot (WB) was carried out for the qualitative assessment of the expression behavior of on a protein level.

**Results:**

The WST-1 test showed a significant inhibition of viability in MFC-7 cells after 72 h at 10 µg/ml. The WB demonstrated a significant quantitative decrease of ERβ at protein level with MTE concentrations of 10 µg/ml. In contrast, the PCR did not result in significant downregulation of ERβ.

**Conclusion:**

MTE decreases the cell viability of MCF-7 cells and furthermore leads to a decrease of ERβ at protein level.

## Introduction

Breast cancer remains the most prevalent cancer among women, with approximately 2.1 million women diagnosed each year, and it is the leading cause of cancer-related deaths in women [1]. Once metastatic disease is diagnosed, the prognosis is often poor, with a median overall survival of only 2 to 3 years, and a 5-year survival rate of 25% [2]. Therefore, there is a pressing need to investigate the potential of widely consumed dietary substances for breast cancer prevention and as therapeutic agents, including plant-derived remedies.

Tea, particularly matcha tea (*Camellia sinensis*), has gained global popularity as the second most common beverage after water [3]. Unlike regular tea, matcha tea is harvested and manufactured through a complex process that involves grinding the leaves into a fine powder, resulting in higher substrate concentrations when mixed with hot water. Matcha tea is distinguished by its high catechin concentration, with epigallocatechin gallate (EGCG) being the most abundant at 90% [4].

Laboratory experiments, animal models, and epidemiological research have suggested anti-cancer properties of EGCG, particularly in estrogen receptor-alpha (ERα) positive breast cancer cells, where it has been shown to inhibit growth by reducing estrogen receptor-beta (ERβ) abundance and increasing p53 and p21 levels in a dose-dependent manner [5].

Furthermore, EGCG has been reported to affect cell cycle and proliferation through various mechanisms, including inhibition of matrix metalloproteinases, Wnt signaling (wingless/integrated protein signaling), methylation, and induction of peroxisome proliferator-activated receptors (PPAR) [6–10].

ERβ was discovered in 1996 and is relevant in hormone-dependent breast cancer, as its expression of over 10% has been shown to be a significant predictor of better clinical outcomes in women treated with tamoxifen. Additionally, ERβ-positive breast cancer is associated with a better prognosis compared to ERβ-negative tumors [11]. Despite previous studies focusing on the alteration of estrogen receptor-alpha by EGCG, specifically investigating the effects of matcha tea extract (MTE) on ERβ in MCF-7 breast cancer cells is lacking. Therefore, in this paper, we aimed to analyze the effects of MTE on ERβ expression in MCF-7 breast cancer cells to elucidate its potential therapeutic effects in hormone-dependent breast cancer.

## Materials and methods

### Cell cultivation and cell stimulation

MCF-7 cells were chosen for their hormone receptor-positive attributes, reflecting the luminal breast cancer subtype. Their estrogen and progesterone receptor presence aligns with our hormone-related focus. Our selection was guided by their relevance to our objectives, well-documented status, and specific interest in MTE's impact on this hormone receptor-positive subtype. Additionally, our choice of MCF-7 cells was influenced by the existing data on green tea phenol's interaction with ERα, while information about ERβ interactions remains limited.

MCF-7 cells were cultured on an 80% monolayer in a cell culture bottle. Dulbecco's modified Eagle medium (DMEM; 3.7 g/l NaHCO3, 4.5 g/l d-glucose, 1.028 g/l stable glutamine, and sodium pyruvate; Biochrom, Berlin, Germany) supplemented with 10% heat-inactivated fetal calf serum (FCS; Biochrom, Berlin, Germany) was used for cultivation. The cells were incubated with atmospheric concentrations of CO_2_ of 5% at 37 °C and were then trypsinized and counted for further use.

### Preparation of the matcha tea extract

The matcha tea extract was commercially purchased (Houjo Matcha Tea, harvested in Hoshino, Yame prefecture, Japan). MTE was prepared as described in the individual tests below.

### Water-soluble tetrazolium assay (WST-1 assay)

MCF-7 cells were cultured in a 96-well plate at a density of 10,000 cells per well in 50 µl of DMEM with 10% FCS. After 4 h, the medium was changed to DMEM without FCS. FCS can contain substances such as growth factors, hormones, vitamins, and transport proteins, which can potentially influence cell proliferation and maintenance. The use of DMEM without FCS helps to reduce this influence on the experimental setup [12, 13]. The cells were further incubated for 12 h.

MTE (27.1 mg) was dissolved in 100 µl of pure ethanol and then diluted 1:1000 with DMEM without FCS. The solution for the control group was diluted in the same manner without adding MTE. Next, different quantities of MTE solution and DMEM without FCS were added to achieve different concentrations (5 µg/ml and 10 µg/ml). A total of 100 µl was added per well. For the control groups, the control solution and DMEM without FCS were pipetted into each well instead of the MTE solution. Incubation was carried out for 72 h.

After incubation, the WST-1 reagent (water-soluble tetrazolium, 4-[3-(4-iodophenyl)-2-(4-nitrophenyl)-2*H*-5-tetrazolio]-1,3-benzenedisulfonate; Sigma-Aldrich, St. Louis, MO, USA) was added. The WST-1 reagent can be used to detect the activity of mitochondrial succinate dehydrogenase, which cleaves the tetrazolium salt to formazan. After 30 min of incubation, the viability of the cells was measured using a multiwell spectrophotometer (wavelength: 420–480 nm). In this study, the approach to sample size and experimental design involved a synergy of independent measurements and technical replicates, thereby facilitating a comprehensive evaluation of the experimental outcomes. The experiment was performed three separate times with three technical replicates, constituting distinct instances of the entire process. This practice minimized the potential influence of varying conditions or external factors, lending credibility to the observed effects.

### PCR

Cell incubation was conducted in a 12-well plate with a density of 500,000 cells per well for 4 h using 500 µl of DMEM with 10% FCS. Subsequently, the medium was changed to 500 µl of DMEM without FCS, and the MCF-7 cells were further incubated for 12 h. For the preparation of MTE solution, 27.1 mg of MTE was dissolved in 100 µl of pure ethanol, and then diluted at a ratio of 1:1000 with DMEM without FCS. The solution for the control group was prepared in the same manner without adding MTE.

To obtain different concentrations (5 µg/ml and 10 µg/ml), various amounts of MTE solution and DMEM without FCS were added, totaling 500 µl per well. In the control group, the control solution and DMEM without FCS were added to each well instead of the MTE solution. This was followed by a 2-h incubation period. Since changes in mRNA levels can occur within hours after stimulation, and the half-life of mRNA is also in the range of hours, a 2-h incubation time was chosen. After incubation, excess liquid was removed, and the wells were rinsed with phosphate-buffered saline (PBS). RA-1 buffer (Macherey–Nagel, Düren, Germany) was added to lyse the cells.

RNA isolation was performed using NucleoSpinRNAII (Macherey–Nagel, Düren, Germany), and reverse transcription of the RNA was carried out using the High-Capacity cDNA Reverse Transcription Kit (Thermo Fisher Scientific, Waltham, MA, USA) with 10 ng of RNA. The temperature protocol included phases of 10 min at 25 °C, 2 h at 37 °C, 5 s at 85 °C, followed by cooling to 4 °C.

For the PCR assay, each well was prepared with 10 µl of TaqMan^®^ Universal PCR Master Mix 2X (Thermo Fisher Scientific), 8 µl of distilled water (treated with 0.1% diethyl pyrocarbonate), 1 µl of TaqMan^®^ Gene Expression Assay 20X (Thermo Fisher Scientific; Target: ACTB, Assay ID Hs999903_m1; Target: ESR2, Assay ID Hs01100357_m1; primer sequences not provided by the manufacturer), and 1 µl of cDNA sample. The PCR assay was performed using the ABI Prism 7500 Fast (Thermo Fisher Scientific).

Thermal cycling for the PCR assay was conducted as follows: the reaction started with an initial denaturation step at 95 °C for 20 s, followed by 40 cycles of amplification at 95 °C for 3 s, and then 60 °C for 30 s. The results were analyzed using the comparative 2−ΔΔCT method [14], with β-actin serving as the endogenous control for the calculation of ΔCT values. As previously implemented in the WST-1 assay, a total of three measurements were conducted, each accompanied by two technical replicates. For RNA isolation, NucleoSpinRNAII kit (Macherey–Nagel, Düren, Germany) was used, and reverse transcription of the RNA was carried out using the High-Capacity cDNA Reverse Transcription Kit (Thermo Fisher Scientific, Waltham, MA, USA) with 10 ng of RNA. The temperature protocol included phases of 10 min at 25 °C, 2 h at 37 °C, 5 s at 85 °C, followed by cooling to 4 °C.

### Western blot

**Fig. 1:**
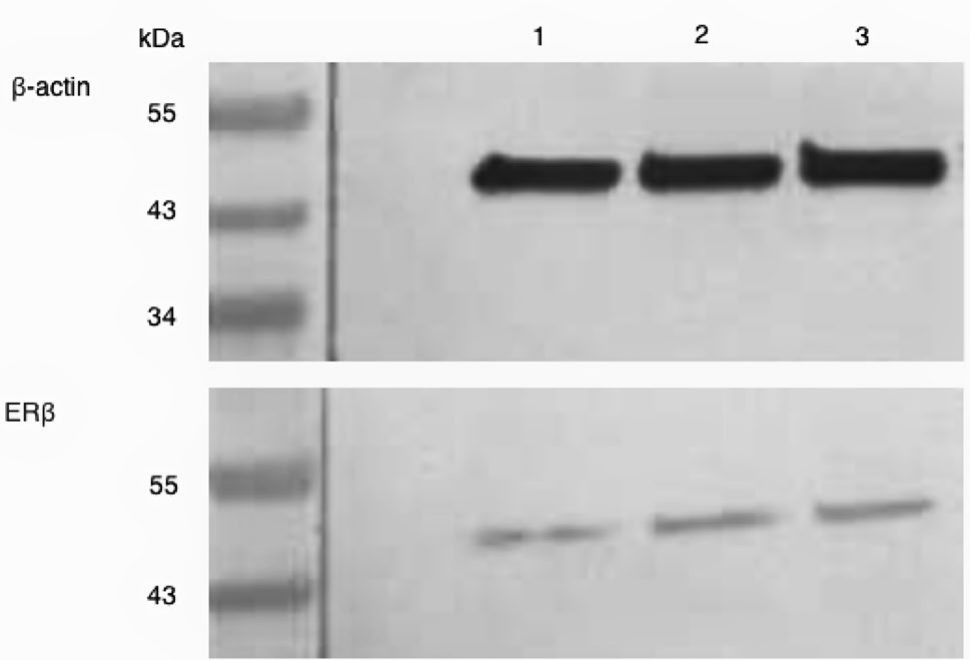
This figure provided exhibits an exemplar of a western blot membrane subsequent to stimulation with varying concentrations of MTE or the control group, following incubation with β-actin and ERß antibodies. The respective bands are numbered as follows: (1) control group, (2) 5 µg/ml, and (3) 10 µg/ml

MCF-7 cells were cultured in 1000 µl of DMEM with 10% FCS in a 12-well plate at a density of 500,000 cells per well. After 4 h, the medium was changed to 1000 µl of DMEM without FCS, and the cells were incubated for an additional 12 h. To prepare the MTE (20 mg) solution, it was dissolved in 100 µl of pure ethanol and then diluted in a ratio of 1:1000 with DMEM without FCS. The control group was prepared in the same way without adding MTE. Test groups with desired concentrations of 5 µg/ml and 10 µg/ml were achieved by pipetting different amounts of the MTE solution and DMEM without FCS, resulting in a total volume of 1000 µl per well. For the control group, 250 µl of control solution and 750 µl of DMEM without FCS were added to each well instead of the MTE solution. The cells were then cultured for 48 h and rinsed with phosphate-buffered saline (PBS). To lyse the cells, 200 µl of a buffer solution containing a 1:100 dilution of protease inhibitor (Sigma-Aldrich, St. Louis, MO, USA) in RIPA buffer (radioimmunoprecipitation assay buffer; Sigma-Aldrich) was added to each well. The cells were then incubated for 30 min at 4 °C and centrifuged to obtain the supernatant for Bradford protein assay. The proteins were separated by SDS-PAGE based on their molecular weight and transferred onto a polyvinylidene fluoride (PVDF) membrane (Merck Millipore, Darmstadt, Germany). The PVDF membrane was blocked for 1 h with a 1 × casein solution (Vector Laboratories, Burlingame, CA, USA) to prevent nonspecific binding of antibodies. The primary antibodies used as endogenous controls were anti-β-actin (clone AC-15, mouse IgG; Sigma-Aldrich Co., St. Louis, Missouri, USA) and anti-ERß (polyclonal IgG, rabbit, Abcam, Cambridge, UK), which were diluted in a 1 × casein solution and incubated on the membrane for 16 h at 4 °C. After rinsing the membranes with Tris-buffered saline (TBST), the membranes were incubated with biotinylated anti-rabbit IgG antibody and ABC-AmP reagent (VECTASTAIN ABC-AmP Kit for rabbit IgG, Vector Laboratories) according to the manufacturer's protocol. The specific bands on the membrane were visualized using the BCIP/NBT chromogenic substrate (Vectastain ABC-AmP Kit, Vector Laboratories) and detected with the Bio-Rad Universal Hood II (Bio-Rad Laboratories, Hercules, CA, USA). The intensity of the color in the ERβ bands was quantified by comparing the clustered pixels to the β-actin bands using the Bio-Rad Quantity One software (Bio-Rad Laboratories). An example of a western blot is shown in Fig. 1. The western blots were repeated nine times for statistical analysis.

### Statistical analysis

The statistical programming environment R, version 4.0.2 [15], was utilized for data processing and statistical analysis. *p *values less than 0.05 were considered statistically significant. Normality of distribution was assessed using Shapiro–Wilk tests. Due to the distinct experimental designs, different tests were employed to compare the experimental groups and control groups. For the WST-1 and PCR assay, paired *t *tests were used. For the western blot analyses, a single-factor ANOVA with post hoc test was employed.

## Results

### WST-1 proliferation assay

**Fig. 2:**
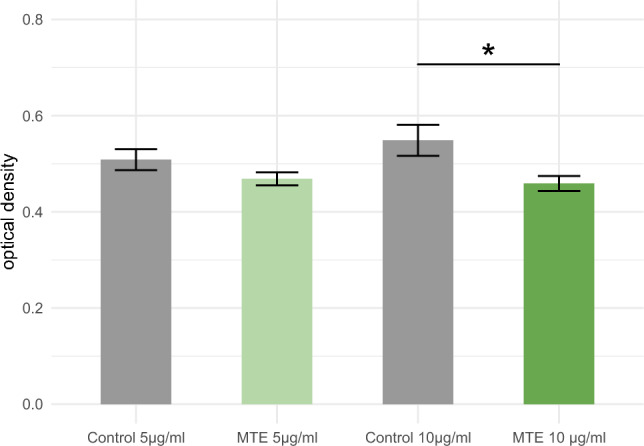
The WST-1 assay was performed on MTE-stimulated MCF-7 cells. The green bars represent the optical density of MCF-7 cells after incubation with different concentrations of MTE (5 µg/ml and 10 µg/ml), while the grey bars represent the control group. The mean ± standard error (SE) is indicated at the top of each bar. MTE resulted in a significant reduction of cell proliferation at the higher concentration, as denoted by one asterisk (*) indicating statistical significance (*p* < 0.05), and the significant results are linked

A WST-1 proliferation assay was conducted to assess the impact of incubation on MCF-7 cellular viability. Statistical significance was observed only at higher concentrations of MTE, with *p *values of 0.074 at 5 µg/ml MTE and 0.017 at 10 µg/ml MTE. The results are presented in Fig. 2.

### PCR for ERß expression on mRNA level

**Fig. 3:**
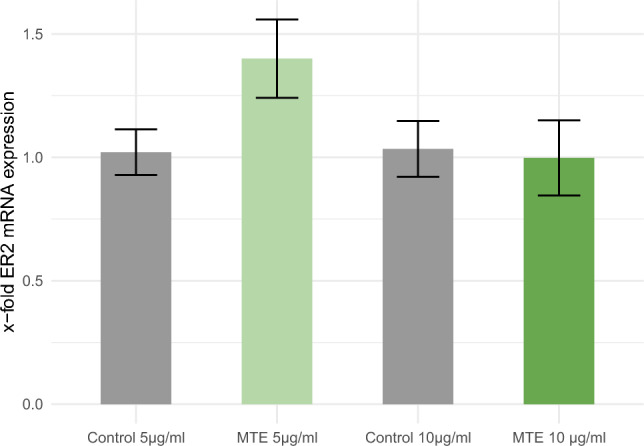
This figure illustrates the relative expression of ERß mRNA in MCF-7 cells following incubation with two different concentrations of MTE (5 µg/ml and 10 µg/ml), as well as the control solution, for a duration of 2 h. The y-axis represents the ratios of stimulated and control expression levels. The mean ± standard error (SE) is indicated at the top of each bar. However, no statistical significance was observed between the groups

The MTE TaqMan^®^ PCR was used to detect changes in ERß mRNA expression in MCF-7 cells after incubation with MTE. The comparative 2−ΔΔCq method was employed for data analysis. However, there were no significant changes observed in the x-fold expression of mRNA in comparison to the control, for both MTE concentrations (5 µg/ml MTE: *p* = 0.091 and 10 µg/ml MTE: *p* = 0.838). The results are depicted in Fig. 3.

### Western blot for ERß on protein level

**Fig. 4:**
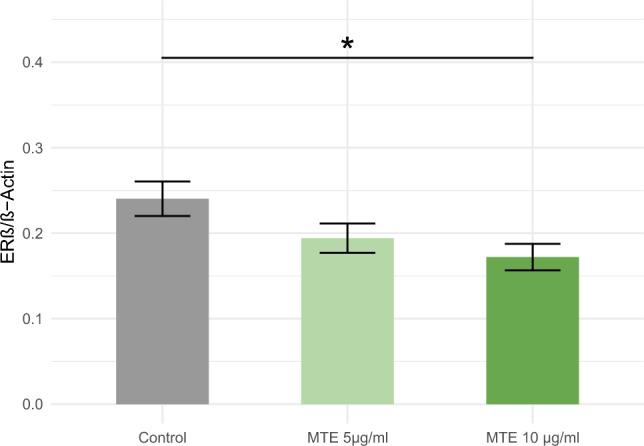
This figure displays the protein expression of ERß in MCF-7 cells following stimulation with varying concentrations of MTE, in comparison to the control solution. The mean ± standard error (SE) is indicated at the top of each bar. Notably, MTE treatment resulted in a significant reduction in ERß expression at the concentration of 10 µg/ml, as denoted by the asterisk (*) indicating statistical significance (*p* < 0.05), and the connecting lines between groups in the figure

The western blot technique was employed to examine alterations in ERß expression at the protein level. Remarkably, a noteworthy decrease in ERß expression was observed solely in the higher concentrations of MTE (5 µg/ml MTE: *p* = 0.233 and 10 µg/ml MTE: *p* = 0.036), as illustrated in Fig. 4.

## Discussion

This study is the first to investigate the effect of MTE on ERβ expression. Our results demonstrate that MTE decreases the viability of MCF-7 cells and leads to a significant decrease in ERβ protein expression.

Generally, phytoestrogens have been found to exhibit a higher affinity for ERβ compared to ERα, as supported by previous research [16, 17]. The binding affinity to ERβ is particularly important, as ERβ signaling has been associated with anti-proliferative and anti-carcinogenic effects, while ERα signaling is linked to carcinogenesis [18, 19]. The loss of ERβ has been correlated with aggressive breast cancers, and ERβ has been recognized as a tumor suppressor gene that regulates ERα-induced proliferation. In a recent study, it was discovered that the phytoestrogen calycosin can upregulate ERβ, leading to diverse effects on downstream cellular signaling pathways. These effects include stimulation of p38 MAPK, suppression of Akt, induction of apoptosis via poly(ADP-ribose) polymerase 1 (PARP-1) cleavage, and inactivation of insulin-like growth factor 1 receptor (IGF-1R) in MCF-7 cells [18, 20].

In contrast to previous studies on other phytoestrogens, our research demonstrates that MTE exhibits dual effects on cellular viability and ERβ expression. This suggests that the decrease in cellular viability may involve mechanisms independent of ERβ downregulation.

Bonuccelli et al. conducted a study elucidating the effects of green tea phenols (GTP) on cancer stem-like cells derived from MCF-7 breast cancer cells. Their findings revealed that GTP preferentially inhibits the proliferative expansion of these cells by suppressing oxidative mitochondrial metabolism (OXPHOS) and glycolytic flux. Consequently, this metabolic modulation leads to a shift in cancer cells toward a more quiescent metabolic state. Additionally, their proteomics analysis identified specific downregulation of mitochondrial proteins and glycolytic enzymes upon GTP treatment. Furthermore, Ingenuity Pathway Analysis (IPA) software analysis indicated a significant impact of MTE on the mTOR signaling pathway [21]. In another study by Liu SM, it was observed that green tea phenols induce dose-dependent apoptosis of MCF-7 cells through mitochondrial pathways [22].

Hence, it is plausible to suggest that the mechanisms of action of MTE are not solely dependent on ERβ modulation. Instead, other cellular mechanisms, as exemplified above, may contribute to the observed decrease in cellular viability.

The western blot and PCR analyses present intriguing contradictions. Notably, the PCR analysis shows a substantial upregulation of ERβ at a concentration of 5 μg/ml, while the western blot data at the same concentration reveal a distinct downregulation of ERβ. The downregulation in the western blot is statistically significant, whereas the upregulation in the PCR analysis lacks statistical significance. This disparity might arise from various factors intrinsic to each technique, including their sensitivities, dynamic ranges, sample variability, technique-related artifacts, and post-transcriptional regulatory mechanisms.

To summarize, our findings indicate that MTE exerts effects on both cellular viability and ERβ expression, suggesting the involvement of mechanisms beyond ERβ downregulation in the observed decrease in cellular viability. The study conducted by Bonuccelli et al. provides evidence of the preferential inhibition of cancer stem-like cells by green tea phenols, involving the modulation of mitochondrial metabolism and glycolytic flux. These cellular mechanisms, along with the impact on mTOR signaling, may play vital roles in mediating the effects of MTE.

The potential clinical application of MTE in the context of breast cancer holds promise as an adjunctive or complementary therapeutic approach. Its observed impact on hormone receptor-related pathways, as indicated by the disparate findings in ERβ expression from western blot and PCR analyses, suggests that MTE might be explored for its modulatory effects on hormone-sensitive breast cancer subtypes. However, further comprehensive investigations, including in vivo studies and clinical trials, are essential to elucidate the exact mechanisms and potential benefits before considering its integration into clinical practice.

## Conclusion

In conclusion, this study provides novel insights into the effects of matcha tea extract (MTE) on cell viability and estrogen receptor-β (ERβ) expression in MCF-7 breast cancer cells. The findings demonstrate that MTE decreases cellular viability and leads to a significant downregulation of ERβ protein expression. These results suggest that the observed effects are not solely dependent on alterations in ERβ, but likely involve other cellular signaling pathways.

Given these intriguing results, it is crucial to unravel the underlying molecular mechanisms responsible for the effects of MTE on ERβ expression and cellular viability. Mechanistic studies, pathway analyses, and in vivo experiments are warranted to provide a more comprehensive understanding of MTE's precise mechanisms of action. Such investigations may have the potential to uncover new avenues for therapeutic interventions and advance our understanding of the complex interplay between phytoestrogens, cellular signaling pathways, and breast cancer progression.

## Data Availability

The datasets used and analyzed during the study are available from the corresponding author on request.
